# Hybrid effectiveness-implementation study designs in sports injury prevention research

**DOI:** 10.3389/fspor.2022.981656

**Published:** 2022-09-20

**Authors:** Hayley J. Root, Monica R. Lininger, Lindsay J. DiStefano

**Affiliations:** ^1^Department of Physical Therapy and Athletic Training, Northern Arizona University, Phoenix, AZ, United States; ^2^Department of Physical Therapy and Athletic Training, Northern Arizona University, Flagstaff, AZ, United States; ^3^Department of Kinesiology, University of Connecticut, Storrs, CT, United States

**Keywords:** implementation science, preventive training programs, injury prevention program, FIFA 11, dissemination

## Abstract

Despite vast evidence supporting the effectiveness of lower extremity injury prevention programs in a variety of sport settings, age groups, and levels of competition, there is limited evidence on implementation strategies that positively impact the feasibility, scale-up and sustainability of such programs. Sport-related injury prevention is affected by the research-to-practice gap, a pervasive issue in healthcare, where high-quality experimental research is not used in routine clinical practice. An intervention shown to be efficacious in a controlled environment, such as a lab or in a field-study conducted by scientists, will demonstrate a decline in benefit when implemented in the intended clinical setting. Real-world considerations, such as foundational knowledge and training, time constraints, or end user motivation, influence the quality and consistency of implementation. Acknowledging and addressing implementation barriers in a systematic way is essential to promote effective program dissemination. Study design methods that measure both clinical effectiveness and implementation strategies need to be identified. Hybrid effectiveness-implementation designs simultaneously measure both an intervention's effect on clinical outcomes as well as critical information related to implementation strategy; however these study designs are not frequently utilized. The purpose of this mini-review is to describe: the basics of hybrid designs, rationale for using hybrid designs, and examples of how these designs could be used in athletic healthcare injury prevention research.

## Introduction

Evidence-based practice (EBP) is the integration of best research evidence, clinician expertise, and patient values to drive clinical decision-making (Sackett et al., 1996; Steves and Hootman, 2004). Unfortunately, <20% of best research evidence is integrated into routine clinical practice and this process takes an estimated 17 years (Morris et al., 2011; Hanney et al., 2015). This research-to-practice gap between empirical evidence and what is done in clinical practice often contributes to racial/ethnic, socio-economic, or other disparities in health outcomes (Shelton et al., 2020; Weiner et al., 2022). Understanding where and why implementation fails will help promote the uptake of evidence-based practices and improve patient-centered care.

Real-world use of interventions can fail for many reasons. Interventions that were tightly controlled with high-levels of internal validity to determine efficacy under a specific set of circumstances may not be robust once necessary adaptations are made to accommodate the realities of different settings. Previous research has grouped factors that influence implementation into patient, provider, innovation, structural and organizational factors (Chaudoir et al., 2013). For example, an efficacious intervention may fail in clinical practice because: the patient is an inappropriate fit, time constraints within the provider's role prevent adding or changing daily tasks and/or the organization culturally does not promote the use of a given intervention (Chaudoir et al., 2013; Medlinskiene et al., 2021). The multitude of factors contributing to sub-optimal implementation and the disproportional research focus on the external validity of interventions deeply impacts the adoption, sustainability, and scale-up of best practice evidence (Glasgow et al., 2019). As such, strategies to systematically track and measure the context of a setting, the implementation strategy used to encourage use of a particular evidence-based intervention, and any adaptations applied to the intervention based on the context are warranted.

Specifically in the lower extremity sport injury prevention literature, there is an abundance of evidence demonstrating the efficacy of researcher-led and effectiveness of closely monitored, coach-led preventive training programs in reducing injury risk metrics (Ardern et al., 2018; Arundale et al., 2018; Padua et al., 2018). However preventive training programs are not a part of routine sport practices across multiple populations (Joy et al., 2013; Norcross et al., 2016; Donaldson et al., 2018; Dix et al., 2021). More systematic evaluations of context and end-user behaviors are needed to address this critical gap between demonstrated efficacy and real-world implementation and sustainability (Benjaminse and Verhagen, 2021).

There are many theories, models, and frameworks designed to help investigators systematically address implementation questions (Owoeye et al., 2020). Of note, the Translating Research into Injury Prevention Practice outlines a stepwise approach that parallels the common scientific pipeline of first understanding injury mechanisms, then developing prevention strategies, testing those strategies in ideal conditions, and then evaluating the prevention strategy with end users in the implementation context (Finch, 2006). A hybrid-effectiveness study would essentially conduct different stages of this framework simultaneously, allowing investigators to start moving evidence into practice faster.

Hybrid studies blend effectiveness study aims and implementation strategy study aims to promote more rapid translation of evidence into real-world practice while considering various contexts and end users (Curran et al., 2012; Landes et al., 2019). Promoting hybrid studies in athletic healthcare will address the research-to-practice gap that hinders many areas of healthcare. Therefore, the purpose of this mini-review is to describe the basics of hybrid designs, rationale for their use, and to provide examples of how these designs could specifically be used in sports injury prevention research.

## Basics of and rationale for hybrid effectiveness-implementation designs

**Figure 1:**
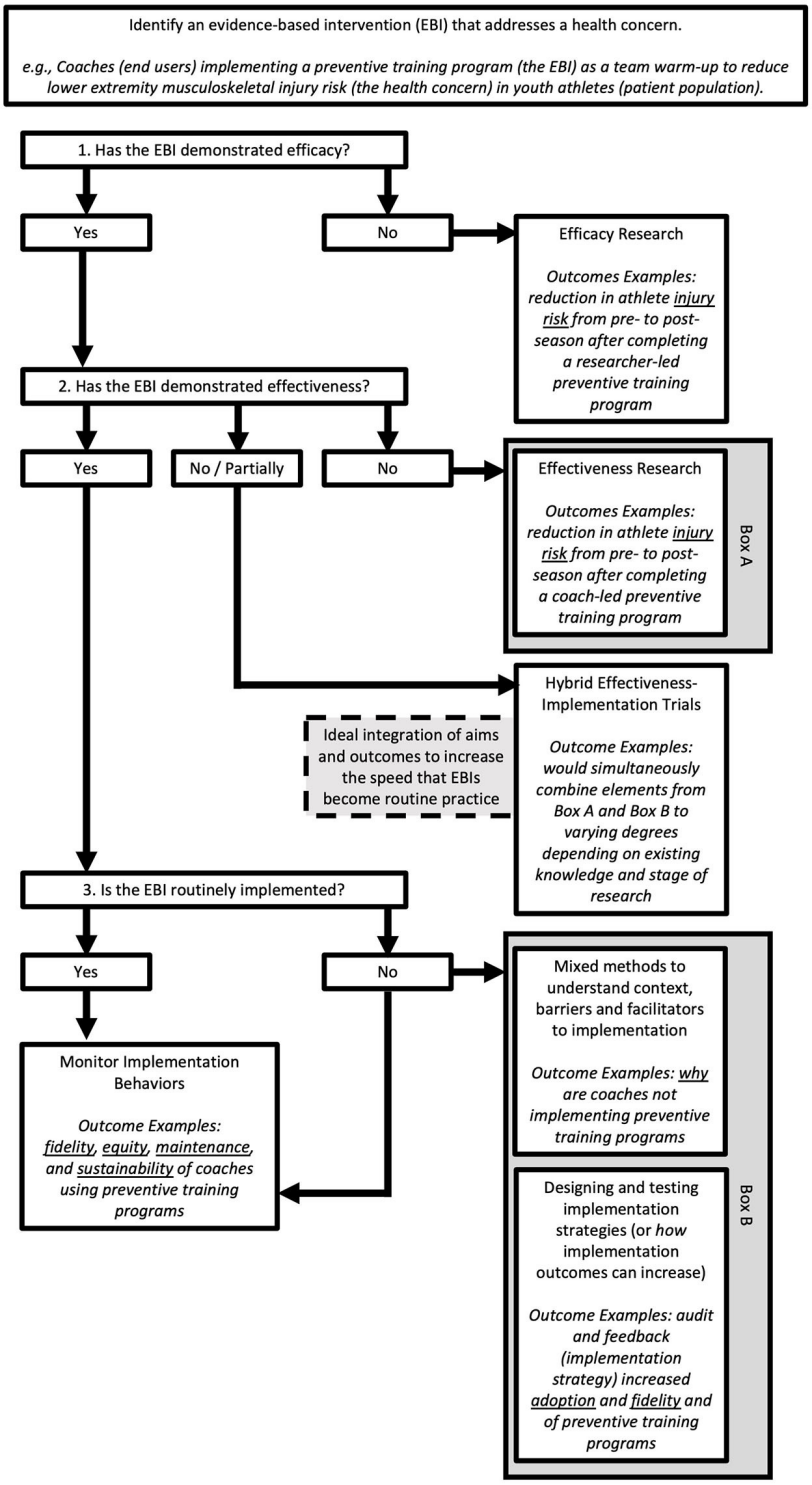
Illustration of the flow of empirical evidence from efficacy to implementation studies. For a given evidence-based intervention (EBI), three pertinent questions should be asked to determine what type of study design could be applied. First, has the EBI demonstrated efficacy in the literature? If no, this is where scientific investigation should begin. If yes, the next question is if the EBI has demonstrated effectiveness? If no, scientific inquiry can begin at this phase (Box A). If yes, then determine if that EBI has been incorporated into routine practice by end-users. For EBIs that are routinely implemented, continued monitoring of implementation outcomes is advised. For EBIs that are not routinely implemented, a variety of approaches can be used (Box B) to better understand context to then inform implementation studies. Hybrid designs combine elements from Box A and Box B for an integrated study approach.

The progression of empirical evidence from discovery in basic science to improvements in public health outcomes is not a linear or unidirectional process. However, it can be helpful to understand how research translation has previously been conceptualized in a linear fashion in order to identify where hybrid designs may be most useful. Figure 1, adapted from previous work and applied to sports injury prevention research (Lane-Fall et al., 2019; Wolfenden et al., 2021), illustrates the progression of research inquiry from efficacy to implementation monitoring and where hybrid designs fit in this process. Key terms are operationalized below.

Efficacy refers to the “performance of an intervention under ideal and controlled circumstances” (Singal et al., 2014). Efficacy trials prioritize high-levels of internal validity to optimize the ability to find an intervention effect. Effectiveness is an intervention's “performance under ‘real-world' conditions” (Singal et al., 2014). Effectiveness trials have more external validity and the end users are implementing a given intervention.

Implementation science seeks to “generate evidence to explain and predict translation of research results and [evidence-based interventions] into practice settings to improve public health and to yield effective methods [to support such translation]” (Weiner et al., 2022). Implementation studies measure how well an intervention is translated into clinical practice. At this point in research translation, the focus is not necessarily on the intervention itself but rather the strategies used to implement a given intervention. Examples of implementation outcomes are adoption (e.g., the number, proportion, or representativeness of settings and providers who employ the intervention) (Weiner et al., 2022) and fidelity (the extent to which an intervention is implemented as designed) (Weiner et al., 2022).

Patient-level outcomes (e.g., strength or injury risk metrics) are not usually included in implementation study designs, where outcomes would look more broadly at global penetration or sustainability of an intervention. However, to enhance implementation there may be adaptations to the intervention itself to improve its acceptability (perception among stakeholders that the intervention is agreeable) and feasibility (extent to which an intervention can be successfully carried out within a given setting) based on context-specific characteristics. The disconnect between studies that aim to evaluate patient-level outcomes and studies that evaluate implementation outcomes is problematic, as it is challenging to discuss how clinical outcomes are impacted by adaptations to interventions without measuring both simultaneously within a study. Traditionally, effectiveness and implementation research has been siloed and sequential. Efficacy studies are typically published first, followed by effectiveness trials, and then implementation research is pursued to evaluate how well the effective intervention is implemented in routine practice and how to improve such implementation. The staged approach from efficacy to implementation is slow and information gleaned at each stage may not be useful, as contexts change more rapidly than work can be published and built upon. Strategies, such as hybrid designs, exist to support varying degrees of outcome integration to improve the speed of translation and utility of best practice evidence (Figure 1) but these strategies are not typically used in athletic healthcare research.

**Figure 2:**
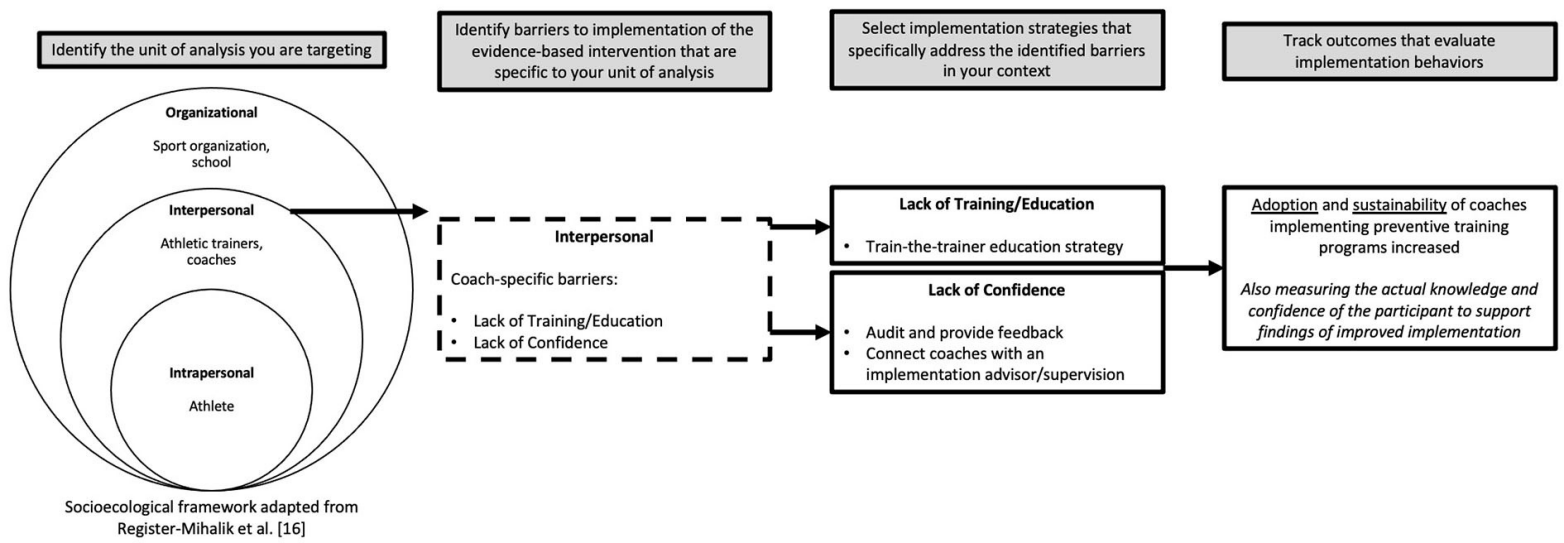
Planning process example of identifying context-specific barriers at multiple levels, choosing implementation strategies that directly address chosen barriers, and then measuring implementation outcomes based on the strategies.

For successful translation of an intervention into clinical practice, researchers and clinicians must determine which interventions work for whom, when, and under what circumstances. As such, discriminating an intervention's core components, or the elements of an intervention that make it successful, from components that could be adapted is necessary. Implementation strategies chosen for an intervention should address context-specific barriers at multiple levels (e.g., individual, organizational) (Register-Mihalik et al., 2017), and any adaptations made to either the intervention itself or its implementation strategy should be specific and explicit to enhance future replicability (Figure 2).

A hybrid effectiveness-implementation design evaluates both the clinical effectiveness of an evidence-based intervention and the implementation strategies selected. There are three main sub-types of hybrid designs with varying degrees of emphasis on the clinical effectiveness or implementation strategy (Table 1).

**Table 1:** Hybrid study design sub-types and examples of application to lower extremity injury prevention research.

**Hybrid design**	**Study purpose**	**Hypothetical application to lower extremity injury prevention research**
Type I	Test the clinical effectiveness with a secondary aim to gather information on the implementation strategy	The primary goal is to evaluate the clinical effectiveness of the preventive training program (PTP) but data collected on a single implementation strategy could inform future comparisons and efforts. ∙ The primary focus evaluates the effectiveness of a lower extremity preventive training program (PTP) to reduce injury risk in youth soccer athletes. ∙ The secondary focus would evaluate if a pre-season educational workshop increased the number of coaches who chose to adopt the PTP.
Type II	Equal focus on the clinical effectiveness and the implementation strategy	Researchers might qualitatively interview key stakeholders at youth soccer organizations (e.g., athletes, coaches, parents and administrators) to understand the context and common barriers to implementation. The information collected could inform a generic implementation strategy. Researchers might go on to randomize organizations and half of the organizations might be more heavily involved in further tailoring their intervention and implementation strategy. ∙ The primary effectiveness outcome could be an athlete injury risk metric from pre- to post-season. ∙ The primary implementation outcome is fidelity to the PTP comparing the tailored organizations to the generic implementation organizations.
Type III	Test the execution of the implementation strategy with a secondary aim to evaluate clinical effectiveness	The primary goal is to compare implementation strategies but patient outcomes are useful to compare to any adaptations made to the intervention or implementation strategy based on context. ∙ The primary outcome is coach fidelity to the intervention comparing a passive dissemination of education materials to an audit-and-feedback implementation strategy. This design could be helpful to compare implementation strategies that have different time and financial costs associated. ∙ The clinical effectiveness outcome might still be athlete injury risk. While this is a lower priority for this study design because global clinical effectiveness of PTPs has already been established, continuing to link patient-level outcomes is useful to potentially explain the impact of any adaptations made to accommodate the implementation strategy.

### Type I

For Type I hybrid designs, the primary focus of the study is to test the clinical effectiveness of an intervention while a secondary aim is to gather information on context for real-world implementation strategy. In this instance, preliminary data related to possible barriers and facilitators to real-world implementation or an evaluation of problems that arose during the study that may be important for future translation are collected. A Type I hybrid design may be appropriate if there is strong existing efficacy data and use of the intervention is supported and needed in a different type of population (Broder-Fingert et al., 2018).

### Type II

In a Type II hybrid design there is an equal focus on the clinical effectiveness and the implementation strategy. In this instance, effectiveness data may be yielding lower outcome change than in efficacy trials, so a closer examination of implementation strategy or strategies is necessary. A Type II hybrid design would be appropriate if there is strong evidence for both the intervention itself as well as strong evidence for the implementation strategy being studied, but the two components are being applied in a novel way—such as in a new population and/or are being studied together for the first time (Hassett et al., 2022).

### Type III

Type III hybrid designs primarily focus on the implementation strategy with a secondary aim to evaluate the intervention effectiveness. A nonhybrid implementation study would strictly evaluate implementation outcomes, such as acceptability and reach of an intervention, after effectiveness data have clearly shown a benefit in a variety of populations and contexts; however, linking clinical outcomes to different implementation strategies is critical to understanding the impact of adaptations.

An example of hybrid designs in other healthcare disciplines is a hybrid III trial conducted in firearm safety. A hybrid III study has a primary emphasis on implementation strategy with a subsequent aim of capturing intervention effectiveness. One study (Beidas et al., 2021) aimed to determine if a less costly and more scalable implementation strategy (implementation strategy A) can change clinician behavior to use an evidence-based firearm safety practice (intervention) compared to a more intensive and expensive facilitation strategy (implementation strategy B). In this example, the arms of the study were implementation strategy A and implementation strategy B to evaluate which strategy could improve implementation of the firearm safety intervention. Outcome measures to evaluate the implementation strategy included: clinician fidelity to the intervention, reach of patients who received the intervention, acceptability, and cost. Outcome measures to evaluate the clinical effectiveness of each implementation strategy included patient-reported firearm storage behavior and youth suicide attempts, death, and unintentional firearm injuries.

Given the need for simultaneous measurement of effectiveness and implementation strategy and the different types of hybrid effectiveness-implementation study designs, it is important to consider how these methods may be applied in injury prevention research.

## Hybrid designs in lower extremity injury prevention research

Sport-related injury prevention research typically follows the staged scientific paradigm described above, where interventions are tested in ideal, controlled circumstances, then real-world circumstances, and finally implementation strategies are assessed for public health impact. Unfortunately, a replication crisis exists at the effectiveness level of science (Peterson, 2021) where outcomes are progressively more diluted as evidence-based interventions are applied to varying populations and contexts (Benjaminse and Verhagen, 2021). However, an improvement in dissemination and implementation science methods (Curran et al., 2012; Brown et al., 2017; Landes et al., 2019) can be applied to lower extremity prevention training programs to enhance real-world impact.

Lower extremity preventive training programs (PTPs) are exercise programs designed to improve neuromuscular control and lower extremity biomechanics to reduce injury risk (Padua et al., 2018). PTPs are typically 15–20 min in length and can be used as a warm-up prior to physical activity. PTP use is supported by a variety of health care groups, such as athletic trainers (Padua et al., 2018), physical therapists (Arundale et al., 2018), and members of the International Olympic Committee (Ardern et al., 2018). There is no clear consensus on a single program, but there are core components that are necessary to incorporate to create an overall effective program (Sugimoto et al., 2016; Trojian et al., 2017; Padua et al., 2018). At a minimum, PTPs should include at least 3 of the following exercise categories: strength, plyometrics, agility, balance, and flexibility, but, most importantly, every PTP should have corrective feedback to ensure participants are performing the chosen exercises correctly in order to optimize neuromuscular changes and reductions in injury risk and rate (Sugimoto et al., 2016; Ardern et al., 2018; Arundale et al., 2018; Padua et al., 2018). Despite strong evidence for the positive benefits of PTPs in a variety of populations and sports, PTPs are not widely used (Joy et al., 2013; Norcross et al., 2016).

While the freedom to modify PTPs can be a strength that allows for a range of population types, sports, and levels of competition to tailor programs for their needs, this leads to exponential intervention and implementation variability, making replicability of positive benefits less predictable. While it is established that PTPs can reduce injury risk and injury rate, implementation strategies that lead to successful maintenance and sustainability are not well understood (Benjaminse and Verhagen, 2021). For example, a stakeholder education strategy to train-the-trainer that is successful in a military or professional soccer organization may not lead to long-term implementation behaviors in a youth basketball organization. There have been studies describing general barriers to PTP implementation (Norcross et al., 2016; Donaldson et al., 2018; Dix et al., 2021), however, it is necessary to systematically explore and report upon implementation strategies that address context-specific barriers. Effectiveness-implementation hybrid study designs, particularly Type II and Type III, would link patient-level outcome data, such as injury risk and rate, with implementation outcomes, such as how well the program was performed or how many stakeholders willingly adopted the program. Understanding if and to what degree implementation strategies are successful in specific contexts, and how that in turn impacts patient outcomes, is critical before PTPs will become part of routine practice in sport.

## Concluding thoughts and future directions

The purpose of this mini-review was to describe the basics of hybrid designs, rationale for their use, and to provide examples of how these designs could be used in sports injury prevention research. Hybrid designs simultaneously measure both an intervention's effectiveness on clinical outcomes and implementation strategy, which promotes more rapid translation of findings into healthcare practice and ultimately addresses the research-to-practice gap.

While we fully support the use of hybrid design approaches, when appropriate, in future research studies, caution is needed due to the level of complexity. Hybrid study designs have multiple levels of analysis (e.g., individual patient clinical outcomes and organization-level randomization of implementation strategies) and an appropriate sample size is necessary at each level to limit risk of a Type II error. As such, statisticians should be a part of the study design planning process. To achieve replicability and understand the potential generalizability of any findings, researchers must operationally define key terms and outcomes. Lastly, due to the complexity of hybrid designs and the multiple layers of analysis and inquiry, it is essential for researchers to clearly report methods and guidelines exist (Pinnock et al., 2017) to help with this elucidation.

## Author contributions

Mini-review conception and design and draft manuscript preparation: HR, ML, and LD. All authors contributed to the article and approved the submitted version.

## Funding

The Athletic Training Program in the Department of Physical Therapy and Athletic Training at Northern Arizona University provided funds for open access publication fees.

## Conflict of interest

The authors declare that the research was conducted in the absence of any commercial or financial relationships that could be construed as a potential conflict of interest.

## Publisher's note

All claims expressed in this article are solely those of the authors and do not necessarily represent those of their affiliated organizations, or those of the publisher, the editors and the reviewers. Any product that may be evaluated in this article, or claim that may be made by its manufacturer, is not guaranteed or endorsed by the publisher.
